# Atomic Layer Assembly Based on Sacrificial Templates for 3D Nanofabrication

**DOI:** 10.3390/mi13060856

**Published:** 2022-05-30

**Authors:** Guangzhou Geng, Zhongshan Zhang, Chensheng Li, Ruhao Pan, Yunlong Li, Haifang Yang, Junjie Li

**Affiliations:** 1 Beijing National Laboratory for Condensed Matter Physics, Institute of Physics, Chinese Academy of Sciences, Beijing 100190, China; genggz@iphy.ac.cn (G.G.); zhangzs@iphy.ac.cn (Z.Z.); chenshengli@iphy.ac.cn (C.L.); panruhao@iphy.ac.cn (R.P.); liyl@cashq.ac.cn (Y.L.); 2 School of Physical Sciences, University of Chinese Academy of Sciences, Beijing 100049, China; 3 Songshan Lake Materials Laboratory, Dongguan 523808, China

**Keywords:** atomic layer deposition, sacrificial templates, 3D nanostructures

## Abstract

Three-dimensional (3D) nanostructures have attracted widespread attention in physics, chemistry, engineering sciences, and biology devices due to excellent functionalities which planar nanostructures cannot achieve. However, the fabrication of 3D nanostructures is still challenging at present. Reliable fabrication, improved controllability, and multifunction integration are desired for further applications in commercial devices. In this review, a powerful fabrication method to realize 3D nanostructures is introduced and reviewed thoroughly, which is based on atomic layer deposition assisted 3D assembly through various sacrificial templates. The aim of this review is to provide a comprehensive overview of 3D nanofabrication based on atomic layer assembly (ALA) in multifarious sacrificial templates for 3D nanostructures and to present recent advancements, with the ultimate aim to further unlock more potential of this method for nanodevice applications.

## 1. Introduction

3D nanostructures with highly-ordered, complex architectures have attracted significant interest in recent years; various devices based on 3D nanostructures have shown excellent functionalities that planar nanodevices cannot achieve [1,2], such as large specific surface area, more spatial dimensions, and multifunctional integration. Especially, these 3D nanostructures can provide additional accessibility and are open to multidimensional interactions, and can also be highly responsive to various external stimuli, which have the potential to develop newer and more innovative applications of all kinds. Due to the unique structural and functional advantages of 3D nanostructures, they are drawing more and more attention in optics [3], electronics [4], photocatalysis [5,6], piezoelectricity [7,8], energy storage devices [9,10], sensing detection [11], and other applications in nanoscience. To consummate and satisfy the kaleidoscopic needs for the fabrication and modulation of nanostructures, dedicated tools and special techniques are usually required, such as electron beam lithography (EBL), focused ion beam (FIB), induced coupled plasma-reactive ion etching (ICP-RIE), metal organic chemical vapor deposition (MOCVD), self-assembly, etc.

Among these conventional techniques, nanofabrication based on atomic layer deposition (ALD) has attracted more attention in recent years, due to its precise conformality and control over materials’ thickness and composition. ALD is based on self-limiting reactions between two gaseous precursors, where the precursors are dosed successively over the growth surface, and achieves the deposition of thin films in a layer-by-layer fashion with atomic-level precision [12,13]. The cyclic and self-limiting nature of ALD enables extremely uniform and conformal films with excellent control over the thickness on virtually any complex substrates [14,15]. Uniformity of ALD film thickness extends not only over flat substrate surfaces but also into narrow holes and trenches. This ability of ALD to make conformal films is called “good step coverage” [16,17]. ALD has emerged as a powerful tool for nanostructure synthesis and functionalization offering unrivalled benefits, especially demonstrating its potential advantages over nanofabrication when combined with various templated structures. Furthermore, ALD enables deposition at low growth temperatures even down to room temperature, which makes ALD suitable for biological and polymer substrates. Up to now, hundreds of ALD processes have been developed to grow an enormous variety of materials, including oxides [18,19], nitrides [20,21], sulfides [22,23], and pure elements [24,25]. For example, representative ALD processes of titanium oxide (TiO_2_), aluminum oxide (Al_2_O_3_), and zinc oxide (ZnO) could be conducted at typical temperature windows of 100–300 °C, and even down to room temperature and as high as 600 °C at certain conditions. Various homoleptic halides, alkoxides, and alkylamides have been widely employed as titanium, aluminum, and zinc precursors. Water is the most common oxygen source, while alternative oxygen sources, such as ozone or plasmas, are sometimes utilized to provide enhanced reactivities in the ALD process. As a result, suitable process parameters should be wisely chosen before utilizing the ALD process. All the advantages of ALD provide great opportunities that are often demanded for today’s advanced devices with dimensions down-scaled to a nanometer level, such as magnetic recording, sensors, and drug delivery in medical treatment [26,27], and, of course, which also offer a new approach to nanofabrication.

The 3D nanostructures in this review infer to the intrinsic structures fabricated through sacrificial templates which are finally removed. Usually, it is quite complex and tough for conventional techniques to fabricate 3D nanostructures with giant controllability and extreme high aspect ratio. Due to the ability to coat or fill complex high-aspect-ratio structures with a wide range of materials and the excellent conformality, ALD has developed into an essential tool for the assembly of novel 3D nanostructures, resulting in an accurate replication of various rigid and soft sacrificial templates, which is collectively referred as the atomic layer assembly (ALA) fabrication method based on sacrificial templates. If 3D structures possess reasonable critical dimensions that are suitable for the ALD method after considering time consumption, and the composed material deposited by ALD is compatible with sacrificial templates, the ALA method is applicable to fabricate the 3D structures.

**Figure 1:**
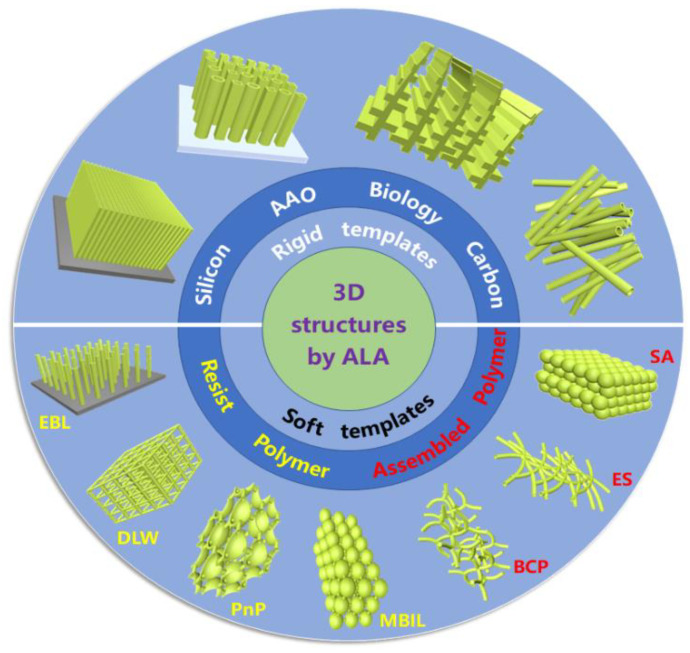
An illustrating summary of the sacrificial templates and obtained typical 3D structures fabricated by atomic layer assembly method. Generally, the sacrificial templates are divided into rigid and soft templates. Typically obtained 3D structures based on ALA method through different sacrificial templates are illustrated in the outer circle of the figure.

Various sacrificial templates have been investigated to fabricate 3D nanostructures through ALA method, and they could be generally divided into rigid and soft templates, as summarized in Figure 1. Here, rigid templates are hard structures, whose stable structures directly determine the size and morphology of 3D nanostructures. Rigid templates could be silicon (Si), anodic aluminum oxide (AAO), carbon, silica sphere, and even biological structures, etc. In the present context, the term “soft” is introduced mainly to differentiate the materials described here from “rigid”. Soft templates could be electron or photoresist polymers, various assembled polymer fibers or membranes, polystyrene (PS) sphere, and so forth.

Generally, the ALA method based on sacrificial templates to obtain the 3D nanostructures includes three steps. Firstly, the sacrificial patterned templates can be fabricated using various advanced nano-techniques. The critical techniques for fabricating Si and AAO rigid templates commonly are lithography combined with etching (dry or wet) and anodization. For soft templates electron or photoresist polymers, electron beam lithography (EBL), direct laser writing (DLW), proximity field nanopatterning (PnP), and multibeam interference lithography (MBIL) are usually utilized. As for assembled polymer templates, block copolymer (BCP), electrospinning (ES), and self-assembly (SA) are regularly employed. Then the films with a certain thickness are deposited on sacrificial templates by ALD for 3D assembly to construct 3D nanostructures. Finally, the residual templates are selectively removed by etching or a heat treatment, and 3D nanostructures shaped by the sacrificial templates can be obtained. Through this ALA method based on sacrificial templates, many kinds of 3D nanostructures could be fabricated, like nanotubes, nanopillars, nanofibers, nanonetworks, inverse opals, and even replication of biological structures [28,29,30,31]. These 3D nanostructures possess full and precise control of size and shapes, considerable reproducibility, and flexible design, making them attractive for a broad range of practical applications.

**Figure 2:**
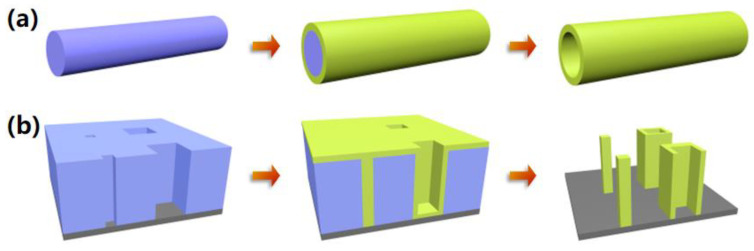
Schematic illustrations of two main fabricating mechanisms for 3D nanostructures based on sacrificial templates by ALA method. (**a**) Mechanism achieving hollow cross-linked 3D nanostructures. (**b**) mechanism achieving arbitrary custom patterns with separated pillars and tubes.

There are two main fabricating mechanisms for the 3D nanostructures based on sacrificial templates by ALA method. As shown in Figure 2a, the first case is that the films are directed coated onto the as-fabricated templates by ALD, fabricating the 3D nanostructures by full replication from the sacrificial templates after removing the templates. The obtained 3D nanostructures keep the same geometry with the outer contours of the templates, generally achieving hollow cross-linked nanostructures. The assembled polymer templates, carbon, biological templates, and resist polymer by PnP and MBIL are regularly geared to the first case, while another fabricating mechanism shows much more flexibilities in the morphology control and could achieve arbitrary custom patterns with separated pillars and tubes. 3D nanostructures fabricated from Si, AAO templates, and resist templates by EBL and DLW usually belong to this second fabricating mechanism. Different to the first mechanism, the caps of the films by ALD are generally eliminated followed by the removal of sacrificial templates, resulting in the separated pillars and tubes with user-defined patterns, as illustrated in Figure 2b. The advantages of the first mechanism are the mass productivity and easy operation, while the second mechanism shows superiority over the flexibility and controllability in custom patterns, showing plenty of applications in various active devices.

In this review, different types of sacrificial templates are highlighted to explore the fabrication of 3D nanostructures based on the ALA method, which consists of five sections. Following the introduction, 3D nanostructures based on rigid and soft templates are systematically categorized and introduced in Section 2 and Section 3. The comparison and applications of 3D nanostructures using various templates are summarized in Section 4. Finally, the conclusion and outlook for the 3D nanostructures through this method are discussed in Section 5.

## 2. Rigid Templates for Fabricating 3D Nanostructures

Rigid templates are defined as opposed to soft templates, and are made of hard materials. Rigid templates have good chemical stability and mechanical rigidity, which are mostly used for the fabrication of nanostructure arrays. A wide choice of rigid templates is available, such as silicon, anodic aluminum oxide, carbon, silica spheres, biological structures, and so forth, which will be discussed later in detail. By an accurate replica of the rigid templates, the dimensions and specifications of 3D nanostructures can be modulated with higher resolution and high aspect ratio. Of course, both achieving and removing these rigid templates often requires a very complex process, and usually the removal process is environmentally unfriendly.

### 2.1. Si Templates

**Figure 3:**
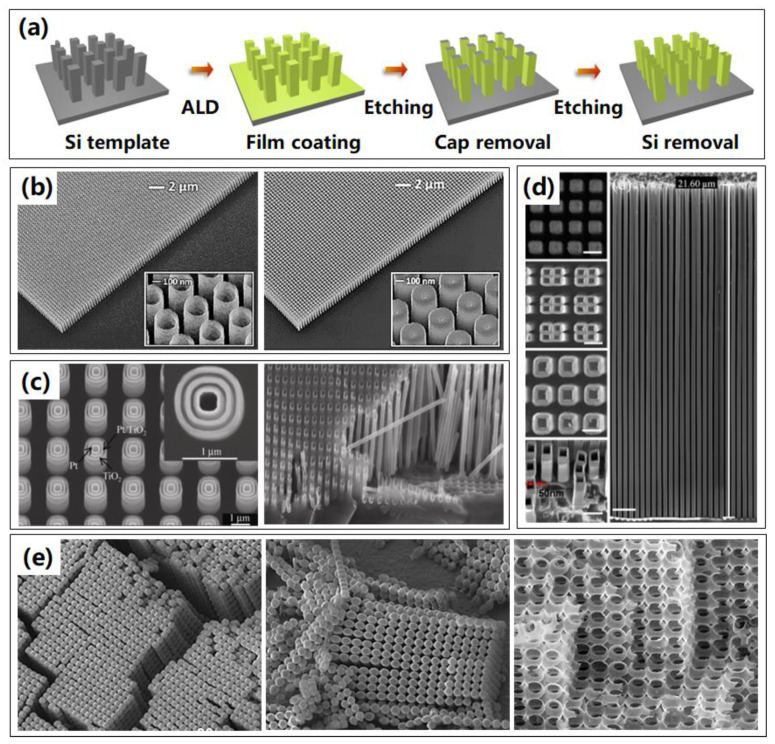
Representative 3D nanostructures fabricated by ALA method through Si templates. (**a**) Process routine of 3D nanostructures based on Si templates by ALA method. (**b**) Titled scanning electron microscope (SEM) images of the 3D metamaterial structures: Al-doped ZnO (AZO) nanotubes and nanopillars. The insets show an enlarged view of the metamaterials. Reprinted from [32], copyright 2017, with permission from Optical Society of America. (**c**) Multi-walled nested nanotube structures with alternating open annular layers after removal via chemical etching of two ALD Al_2_O_3_ sacrificial spacer layers, cleaved Si template highlighting that ALD Pt has infiltrated up to 90 µm deep into the bottom of the porous Si template. Reprinted with permission from Ref. [35]. Copyright 2011 Springer. (**d**) Top view SEM image of the Si nanostructures after Al_2_O_3_ film deposition by ALD, and SEM images of the top, cross-section, and tilted views of the generated Al_2_O_3_ nanotube arrays resulting from the sidewall transfer MacEtch process. Reprinted with permission from Ref. [36]. Copyright 2020 MDPI. (**e**) An array of hollow TiO_2_ micro-pearl chains viewed from the bottom and the side, and enlarged SEM images of TiO_2_ porous network structures. Reprinted with permission from Ref. [37]. Copyright 2008 Springer.

Perhaps the most well-known material to fabricate nanostructures, silicon may be the most widely investigated due to its mature fabrication process. Therefore, it is natural for researchers to fabricate 3D nanostructure replicas by utilizing silicon nanostructures as sacrificial templates. Figure 3a exhibits the fabrication routine of 3D nanostructures by ALA method through Si templates. Firstly, the Si templates are commonly prepared by lithography and etching process, resulting in templates with nanopillars or nanopores. Then the ALD process is applied sequentially and generally followed by a removal of the ALD cap on top of the structure. The last step is silicon template removal, which is done by dry or wet etching, leaving the high aspect ratio 3D nanostructures without distortion. In such a way, 3D nanostructures could be obtained, whose shape, size, and thickness can be modulated with higher resolution and high aspect ratio [32,33,34]. The success of this procedure is heavily dependent on the ability to etch silicon selectively and realize the perfect replication from photoresist patterns to Si templates, without destroying the ALD coatings, which also limits the diversity and resolution in nanostructure for the Si templates.

As shown in Figure 3b, large-area 3D Al-doped ZnO (AZO) nanopillars and nanotubes using a combination of advanced reactive ion etching and ALD techniques were fabricated through sacrificial Si templates [32]. It was found that the last step of Si template removal process was heavily dependent on the selective chemistry of the SF_6_ plasma. It was shown that silicon between AZO structures can be selectively removed with no observable influence on the ALD deposited coatings. Furthermore, complex multi-walled nested TiO_2_–Pt nanotubes in series have been successfully fabricated using microporous Si templates, as shown in Figure 3c (left) [35]. Multilayered nested nanotubes separated by sacrificial spacer layers of TiO_2_ (50 nm)/Al_2_O_3_ (120 nm)/Pt (25 nm)/TiO_2_ (25 nm)/Al_2_O_3_ (120 nm)/Pt (50 nm) were firstly fabricated, followed by removal of the Al_2_O_3_ sacrificial spacer layers and the porous Si template by using NaOH solution, resulting in the novel multi-walled nested structures. These innovative nested nanostructures have the potential to produce novel multifunctional vertically-ordered 3D nanodevices in photovoltaic and sensing technologies. Especially, optimizing the ALD process by using an additional 10 s exposure time, as-formed Pt could infiltrate up to 90 µm deep into the bottom of the porous Si template, achieving extreme high aspect ratio Pt nanotubes in Figure 3c (right).

Furthermore, ultra-high aspect ratio Al_2_O_3_ nanotube arrays with line widths as small as 50 nm, heights of up to 21 µm, and an aspect ratio of up to 420:1 were successfully fabricated, utilizing reliable sidewall transfer low-temperature Au metal assisted chemical etching (MacEtch) process, as displayed in Figure 3d [36]. This technique combined the advantages of the high aspect ratio nanostructure capabilities of the MacEtch with the sidewall transfer process. Such a sidewall transfer MacEtch approach was compatible with well-established silicon planar processes, and has the benefits of possessing a fully controllable linewidth and height, high reproducibility, and flexible design, making it attractive for a broad range of practical applications. Similar high aspect ratio 3D nanostructures were investigated [34], while Bosch process was applied to fabricate the Si template with high aspect ratio nanoholes. As long as complex Si templates could be manufactured, more complex nanostructures could be realized by replication of the complex Si templates. As shown in Figure 3e, 3D macroporous silicon template was firstly fabricated by wet etching method [37], then hollow TiO_2_ micropearl chains and networks were obtained by ALD after removal of Si template. For possible applications, the presented TiO_2_ micropearls are well suited as carriers for drug delivery, whereas the network structures can work as highly porous photocatalytic material.

The advantages of Si templates are the mature fabrication process, custom flexible design, and easy implementation for array fabrication. While the removal of Si templates, in which wet etching is mostly utilized, is usually complicated and contaminative. Furthermore, it is a challenge to preserve exactly the morphology of the as-deposited nanostructures.

### 2.2. AAO Templates

**Figure 4:**
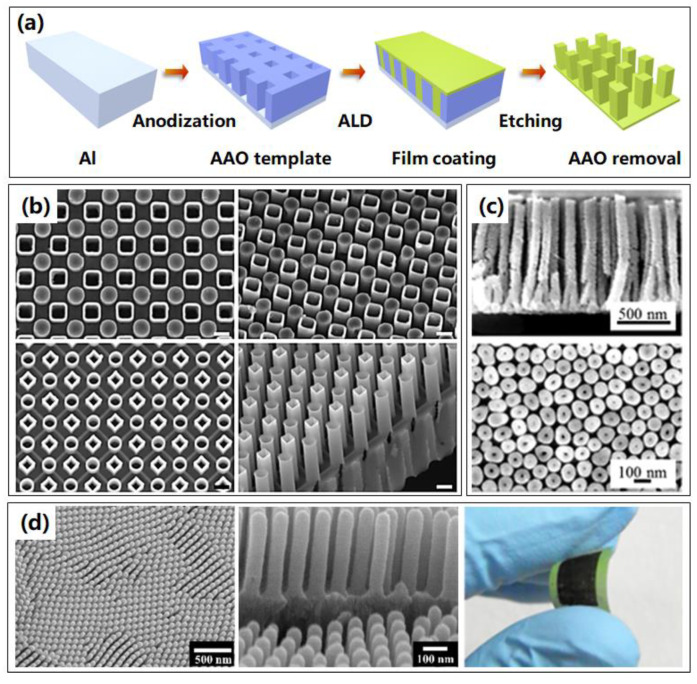
Typical 3D nanostructures fabricated by ALA method through AAO templates. (**a**) Process routine of 3D nanostructures based on AAO templates by ALA method. (**b**) SEM images of representative 3D binary nanostructure arrays. The upper two show nanostructures with nanopillars and nanotubes, and the lower two are nanostructures with all nanotubes. Reprinted with permission from Ref. [40], Copyright 2017 Macmillan Publishers Limited, part of Springer Nature. (**c**) SEM images of 3D ZnO nanowires for sensor applications. Reprinted with permission from Ref. [2], Copyright 2016 American Vacuum Society. (**d**) Overall and enlarged SEM images, and photograph of flexible 3D TiN nanotubes arrays. Reprinted with permission from Ref. [30]. Copyright 2020 MDPI.

Another widely investigated rigid template is the anodic aluminum oxide (AAO) template. AAO is an appealing template to fabricate highly ordered and regular nanostructures, showing advantages including low cost, good compatibility, easy scalability, and flexible controllability, and has been exploited for fabricating various nanostructure arrays [38,39]. As shown in Figure 4a, the fabrication process of 3D nanostructures based on AAO template is practically accordant with Si template, while the AAO templates are usually prepared by anodization method, resulting in templates with various nanopores. Once the AAO templates are prepared, the same following processes as ALD of certain materials and removal of cap and templates are conducted successively. An AAO template possesses significant advantages due to its uniform nanochannels size, tunable pore dimensions, good mechanical strength, and thermal stability. The morphological parameters of the 3D nanostructures, such as the packing density or the aspect ratio, are fully controlled by the geometrical parameters of the AAO template.

Diverse binary or multi nanostructure arrays with high degrees of controllability for each of the sub-components, including material, dimension and morphology, as shown in Figure 4b, were fabricated based on ALA method through an AAO template [40]. This binary nanostructure originated with a distinctive binary-pore AAO template that included two dissimilar sets of pores in one matrix, where the openings of the two sets of pores were towards opposite sides of the template. They also presented proof-of-principle photoelectrodes, transistors, and plasmonic devices made with binary nanostructure arrays using different combinations of materials and morphologies, and demonstrated superior performances compared to their single-component counterparts.

Due to the high surface area, which will enlarge the contact area between structures and reactant, another important application of 3D nanostructures is sensors. A new low-temperature nanowire fabrication process using AAO sacrificial templates was presented [2], which allowed high aspect ratio nanowires to be readily integrated with microelectronic devices for sensor applications. As displayed in Figure 4c, the resulting ZnO nanowire array enhanced the sensor surface area by ~38 times over a conventional flat film, which was supposed to improve capacities of sensors, like sensitivity and response speed.

Flexible devices have achieved remarkable progress over the past years and have become increasingly important to many sectors. Even the AAO template is a rigid template, however, it can also be utilized to fabricate flexible devices. In 2020, the TiN nanotube arrays on flexible substrates were achieved by ALA method through AAO template [30], as shown in Figure 4d. The flexibility of the fabricated nanostructures was investigated using the cyclic bending and releasing method at high curvature. No noticeable cracks or delamination were detected upon operation after 1000 cycles. Meanwhile, delamination and faceted phenomena were not observed after 3000 cycles, implying that this method has a great potential for application in flexible systems as a 3D electrode.

For AAO templates, some problems of complicated post processes are inevitable, and the removal of the AAO template using chemical solvent is a big problem to be compatible with the fabrication of conventional Si-based integrated circuits. The compatibility with other common mature industrial processes needs to be developed further and the environmental concerns should be resolved.

### 2.3. Biological Templates

**Figure 5:**
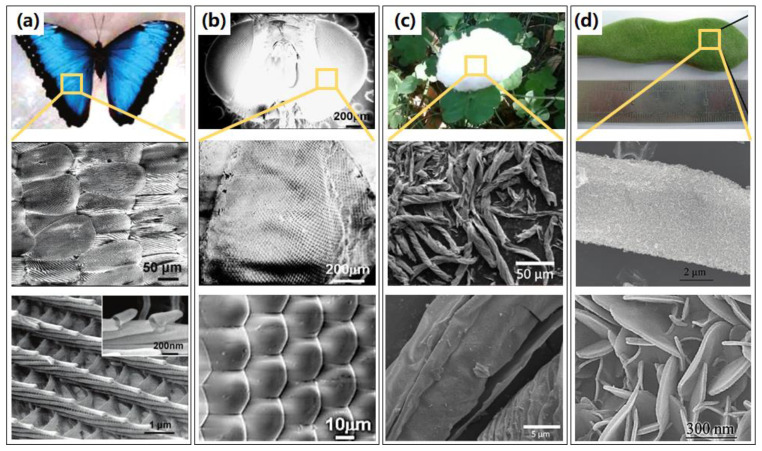
Attractive 3D nanostructures fabricated by ALA method through biological templates, with the photo image of veritable biological template, overall and enlarged SEM images in local area. (**a**) The biological templates of butterfly wings, with overall and enlarged SEM images of the alumina replicas of the butterfly wings. Reprinted with permission from Ref. [31], Copyright 2006 American Chemical Society. (**b**) Veritable biological templates of fly eyes and SEM images of the alumina replica of a fly compound eye. Reprinted with permission from Ref. [41], Copyright 2008 IOP Publishing Ltd. (**c**) Veritable biological templates of cotton, overall, and enlarged SEM images of fibrous Al_2_O_3_ nanotube network. Reprinted with permission from Ref. [42], Copyright 2018 Wiley-VCH Verlag GmbH & Co. KGaA, Weinheim. (**d**) Veritable biological templates of tubular trichome on legume, with SEM images of mixed metal oxides framework obtained using a combination of ALD in situ growth calcination. Reprinted with permission from Ref. [43], Copyright 2009 American Chemical Society.

Apart from the artificial templates discussed above, the rigid templates even show enormous flexibilities in biological structure. Nature provides extensive micro-nanostructures, which could be utilized as templates to fabricate various 3D structures, such as photonic crystals, fiber networks, etc. Compared with other rigid templates, the bio-templates have the advantage of availability in a wide variety of sources, complex structure, nontoxicity, and easy removal, and they have great potential to be used as templates for the synthesis of other materials. A variety of biological templates have been utilized to fabricate 3D nanostructures, such as veritable biological templates of butterfly wings, fly eyes, cotton, and tubular trichome on legume, as shown in Figure 5. The first bio-inspired novel biostructures with the scale of a butterfly wing [31] were replicated through an Al_2_O_3_ coating by a low-temperature ALD process. An inverted 3D biostructure was achieved by removing the butterfly wing template at high temperature, forming a polycrystalline Al_2_O_3_ shell biostructure with precisely controlled thickness, as presented in Figure 5a. Other antireflection nanostructures were fabricated by replicating fly eyes using the same process, as shown in Figure 5b, indicating potential applications in optical coating, sensing, or lens arrays [41]. Furthermore, Tian et al. [42] deposited Al_2_O_3_ on the surface of low-cost cotton fibers, and hollow Al_2_O_3_ fibers were synthesized after the elimination of sacrificial cotton templates, and retained the micro- and macrostructures of the cotton. This low-weight Al_2_O_3_ fiber network with elastic and porous 3D structures overcomes the issues resulting from the 2D rigid Al_2_O_3_ layer and provides a low overpotential and dendrite-free growth of lithium metal, as shown in Figure 5c. Furthermore, a biomorphic mixed metal oxide framework through a legume bio-templated synthesis method by using a combination of ALD-in situ growth-calcination [43], showing potential applications in catalysis and adsorbents, was reported and investigated, as shown in Figure 5d.

Other groups also investigated various nanostructures with remained biological templates, which enriched the feasibility and functionality of biological templates [44,45,46]. The advantages of biological templates are low cost, natural structural diversity, and highly accessible, which show enormous possibilities in commercial applications and deserve to be investigated further. However, the low chemical stability at high temperature, low tolerance for multistep fabrication processes, and weak reusability impede the development of the biological templates.

### 2.4. Other Rigid Templates

**Figure 6:**
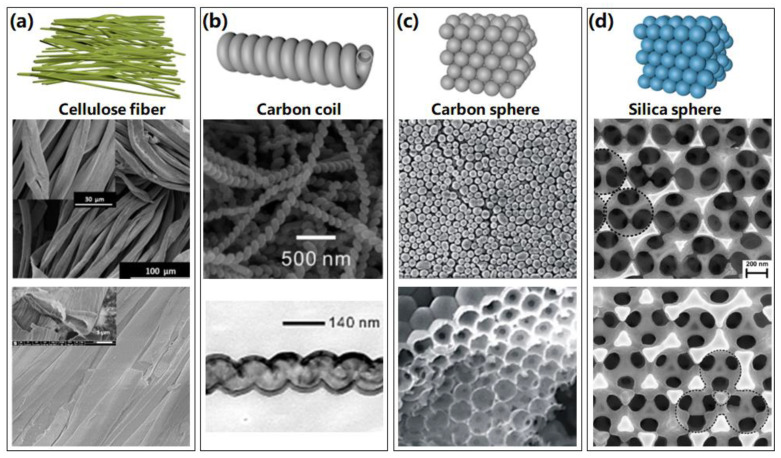
Several special 3D nanostructures fabricated by atomic layer assembly method through cellulose, carbon, and silica templates. (**a**) Schematic images of cellulose fiber templates, SEM images of TiO_2_ fibers on cellulose fibers, and SEM images of Al_2_O_3_ nanofibers after cellulose extraction. Reprinted with permission from Ref. [47], Copyright 2017 American Chemical Society. (**b**) Schematic images of carbon coil templates, SEM image of as-formed helical Al_2_O_3_ nanotube after removal of templates, and TEM image of TiO_2_ helical nanotubes after removal of templates. Reprinted with permission from Ref. [49], Copyright 2010Wiley-VCH Verlag GmbH & Co. KGaA, Weinheim. (**c**) Schematic images of carbon sphere templates, and SEM images of veritable templates of carbon sphere opal and TiO_2_ inverse opal. Reprinted with permission from Ref. [50], Copyright 2020 Elsevier. (**d**) Schematic images of silica spheres templates, and SEM images of TiO_2_ close-packed inverse opals and non-close-packed inverse opals. Reprinted with permission from Ref. [51], Copyright 2006 WILEY-VCH Verlag GmbH & Co. KGaA, Weinheim.

Except the templates discussed above, there are several special rigid templates that could be utilized to fabricate 3D nanostructures, such as cellulose, carbon coil, carbon spheres, and silica spheres, as schematically shown in Figure 6. Ortal et al. [47] deposited TiO_2_ nanofilms by ALD onto cellulose microfibers, leading to the formation of chiral nanofilms with a spatial fibrous structure, which show potential applications in enantioselective areas. The obtained fibers on cellulose fibers and after cellulose extraction are displayed in Figure 6a. Natural cellulose was also investigated to prepare TiO_2_, ZnO, and Al_2_O_3_ nanotube aerogels, resulting in an efficient humidity sensor with a fast response [48].

A forest of 1D anatase TiO_2_ nanoparticle chains was also synthesized based on ALA method using carbon nanotubes as sacrificial templates [52]. The resulting self-supported structure of fully crystallized nanoparticles offers a porous network with a large surface area. As shown in Figure 5c and Figure 6b, carbon nanocoils [49] and nanospheres [50] were also investigated and used as sacrificial templates to prepare distinctive helical nanotubes and inverse opal photonic crystals, respectively, indicating potential applications in sensors, mechanical springs, and photocatalysis areas. Similarly, silica nanospheres [51] were utilized to fabricate inverse opal photonic crystals as shown in Figure 6d, which revealed a potential application in photocatalytic and solar cells.

However, the carbon and silica templates possess the disadvantages of inflexible structure and low variability, which restrict the realization of custom structures and functional expansion based on those templates. With the development of modern techniques, it is believed that more kinds of rigid templates will be fabricated and investigated to achieve 3D nanostructures based on ALA methods.

## 3. Soft Templates for Fabricating 3D Nanostructures

Just like rigid templates, there are numerous types of soft templates, including electron resist polymer, photoresist polymer, and various assembled polymers consisting of block polymer, fiber or membrane, polystyrene (PS) sphere, and so forth. These versatile soft templates can be used in the ALA method and have broad prospects for development in powerful fabrication of multiple nanostructures, which possess a lot of advantages, such as simple process, good flexibility, repeatable simplicity of the process, and environmentally friendly easy elimination of the templates, resulting in diversiform 3D nanostructures with numerous device applications.

### 3.1. Resist Polymer Templates

**Figure 7:**
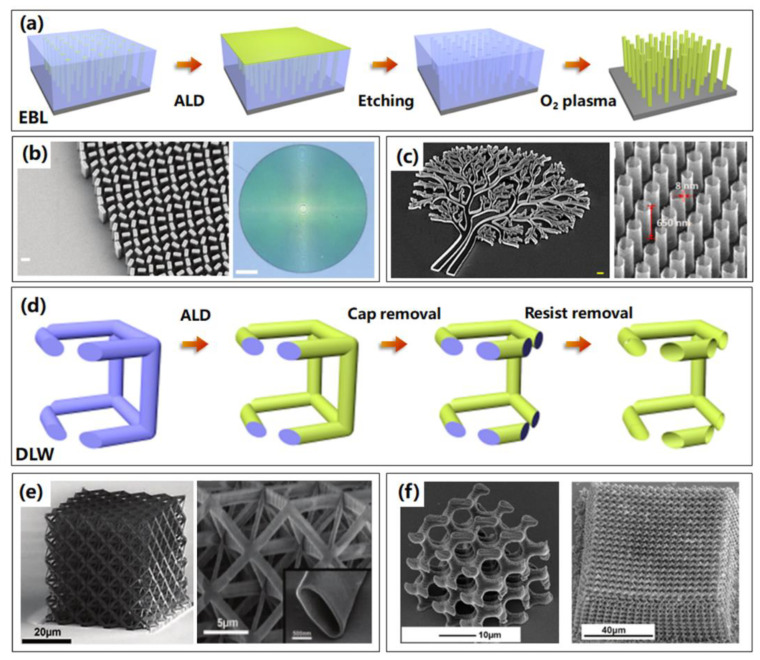
Resist polymer templates by EBL and DLW processes are used to fabricate various 3D nanostructures based on ALA method. (**a**) The illustrated process routine of 3D nanostructures based on electron resist templates through EBL technique. (**b**) SEM and optical images of the fabricated metalens. Reprinted with permission from Ref. [53], Copyright 2016 American Association for the Advancement of Science. (**c**) SEM images of the tree-shaped irregular nanostructures and nanotubes with ultra-high aspect ratio of more than 80:1, based on ALA method through electron resist templates by EBL technique. Reprinted with permission from Ref. [54], Copyright 2021 Elsevier. (**d**) The illustrated process routine of 3D nanostructures based on photoresist templates by DLW technique using ALA method. (**e**) Overall and enlarged SEM images of the overall alumina octet-truss nanolattice through photoresist by DLW technique based on ALA method. Reprinted with permission from Ref. [57], Copyright 2014 American Association for the Advancement of Science. (**f**) SEM images of two kinds gyroid photonic crystals through photoresist by DLW technique based on ALA method. Reprinted with permission from Ref. [58], Copyright 2016 American Chemical Society.

Due to the utilization of direct writing techniques, resist templates, including electron resists and photoresists, maybe the most flexible and controllable templates for 3D nanostructure fabrication based on ALA method. Nowadays, miniaturized conventional systems require the development of custom applications in imaging, displaying, and spectroscopy, especially for 3D nanosystems and 3D nanostructures. As a result, electron and photoresist templates, which could achieve 3D nanostructures by electron beam lithography (EBL) and direct laser writhing (DLW), are proposed and investigated in many fields, and their fabrication processes are illustrated in Figure 7a,d, respectively. Firstly, the electron or photoresists are patterned by EBL or DLW, and then ALD is applied to fill or coat the obtained templates to assemble 3D nanostructures which are confined by templates. Followed by the removal of the cap of the coated template and exposing residual resist polymer, the resist polymer is removed by O_2_ plasma or remover.

In 2016, a novel process for fabricating dielectric metasurfaces through soft electron resist templates based on ALA method was first proposed, which could produce anisotropic, subwavelength-spaced 3D nanostructures with shape birefringence [53]. They patterned the electron resist using EBL firstly, resulting in templates with patterns in reverse of the final metasurface. Similar to the process to fabricate 3D nanostructures as discussed above, ALD-based TiO_2_ films were coated on the template, followed by the removal of TiO_2_ cap and residual resist, high performance 3D nanofin metasurface as a metalens, as shown in Figure 7b, was successfully fabricated and showed widespread applications in laser-based microscopy, imaging, and spectroscopy. This ALA process should be the simplest strategy to realize the complex 3D nanostructures of dielectric metamaterials up to now, which is also capable of achieving any high-efficiency metasurface optical element. Geng et al. developed the ALA method deeper to fabricate 3D nanostructures utilizing electron beam resist, resulting in large-scale arrays of multiple complex 3D nanostructures with high resolution down to nanometers and ultra-high aspect ratio of hundreds. Particularly, an extreme structural nanotube array with ultra-high aspect ratio of more than 80:1 (8 nm wall thickness and 650 nm height) is assembled successfully. The results are displayed in Figure 7c [54].

Creating lightweight, mechanically robust materials has long been an engineering pursuit. Many siliceous skeleton species, such as diatoms, sea sponges, and radiolarians, have remarkably high strengths compared with man-made materials of the same composition, yet are able to remain lightweight and porous [55,56]. As shown in Figure 7e [57], to investigate the lightweight nanostructures, creation of ceramic nanolattices begins with the design and writing of a negative photoresist template using two-photon lithography direct laser writing (DLW), achieving a polymer sacrificial template. Al_2_O_3_ film is then deposited onto the soft template by ALD, so that it coats the entire surface of this 3D skeleton. Then the internal polymer is etched away in O_2_ plasma after the removal of the outermost sides of the coated structure by focused ion beam milling (FIB), resulting in 3D ceramic nanolattice consisting of a network of hollow tubes, which can recover their original shape after compressions in excess of 50% strain. This fabrication method enabled the creation of 3D structures with numerous geometries and custom designs, exhibiting a strong, ultralight, energy-absorbing, and recoverable metamaterial, called a mechanical metamaterial. Using a similar technique based on a photoresist template, gyroid photonic crystals were fabricated successfully, as shown in Figure 7f, which exhibited a complete bandgap in infrared spectroscopy measurements [58].

Due to the fact that it commonly relied on negative resists using two-photon lithography, the 3D nanostructure fabrications based on photoresist are limited [59,60], whose topography and size are restricted. Consequentially, electron resist shows more advantages than DLW photoresist in fabricating 3D nanostructures based on ALA method, which can maximize the virtues of following ALD process in flexible custom topography, precise size control, and more compatibility, further showing giant applications in various optical devices [61,62,63,64,65,66,67,68]. Despite the long-time consumption, the fabrication of 3D structures through resist polymers by ALA method shows incomparable flexibilities and great compatibility with conventional semiconductor processes, showing ability in achieving arbitrary custom pattern and complicated 3D structures with extreme geometries. New techniques combined with ALA method, like nanoprinting and multibeam exposing, should be developed to reduce the time consumption.

**Figure 8:**
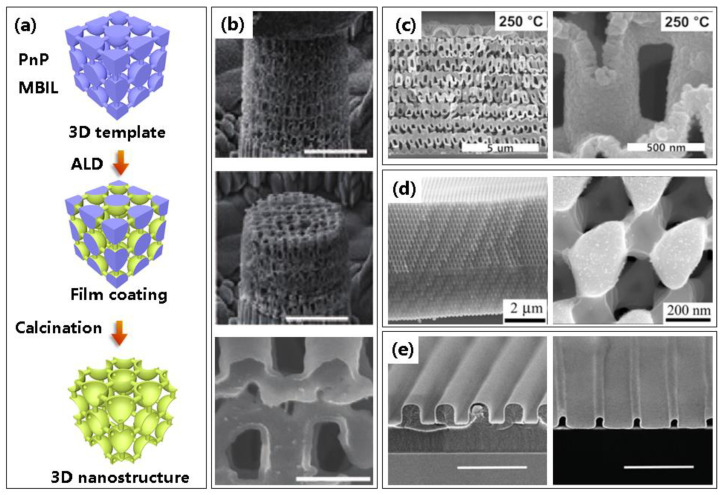
Other photoresist polymer templates by PnP and MBIL processes are used to fabricate 3D nanostructures by ALA methods. (**a**) Illustrated process routine of 3D nanostructures based on photoresist templates by PnP and MBIL techniques. The two techniques commonly share the same fabrication process, except the methods to fabricate resist templates. (**b**) SEM images of Al_2_O_3_ nano-architectures based on PnP technique taken before and after compression tests. And the enlarged SEM images of Al_2_O_3_ nano-architectures. Reprinted with permission from Ref. [72], Copyright 2018 WILEY-VCH Verlag GmbH & Co. KGaA, Weinheim. (**c**) SEM images of 3D ZnO hollow nanostructure deposited at 250 °C after removal of the epoxy template based on PnP method. Reprinted with permission from Ref. [73], Copyright 2020 Elsevier. (**d**) 3D TiO_2_ photonic crystals after removal of the polymeric template by MBIL method. Reprinted with permission from Ref. [74], Copyright 2006 WILEY-VCH Verlag GmbH & Co. KGaA, Weinheim. (**e**) SEM images of polymer grating template by MBIL method with Platinum film by ALD; and free-standing Pt nano-accordions after removal of the template. Reprinted with permission from Ref. [75], Copyright 2016 The Royal Society of Chemistry.

Apart from EBL and DLW, several other techniques could be utilized to pattern resist templates. As presented in Figure 8a, two feasible techniques to pattern photoresist templates are the proximity field nanopatterning (PnP) technique and multibeam interference lithography (MBIL).

PnP is a versatile 3D nanopatterning technique that creates highly ordered 3D nanostructures in photosensitive materials by capturing the 3D light distribution, generated by a phase mask with periodic relief structures. The 3D light intensity distribution, called the Talbot effect or self-imaging effect [69,70], has periodically repeated images generated by interferences of diffracted beams. This method can rapidly fabricate 3D nanostructures through only single exposure over a large area (>1 inch^2^) [71]. As displayed in Figure 8b, hollow-tube-based 3D Al_2_O_3_ nanoarchitectures functionalized as lightweight materials were fabricated in large areas using PnP and ALA process [72]. The first two SEM images show nanoarchitectures taken during compression and after complete unloading, which revealed that the strengths of these nanoarchitecture materials were powerful. The zoomed-in image of the single unit cell is exhibited in the bottom of Figure 8b. Another group [73] reported a truss-like 3D hollow ZnO nanostructure using PnP method that exhibits a drastically improved elastic strain limit while maintaining a piezoelectric coefficient similar to that of single crystal ZnO, showing excellent potential application in enhanced haptic devices, flexible sensors, and energy harvesters. The SEM images of 3D ZnO hollow nanostructures after removal of the epoxy template are displayed in Figure 8c.

Unlike PnP method, multibeam interference lithography (MBIL) is a maskless and practical fabrication technique of periodic microstructures over large areas [76,77]. This technique possesses advantages of low cost, no contamination, and fabricating pattern over a large area (up to mm in diameter), but the variety of the patterns is limited due to the exposure mechanism of MBIL, such as an anisotropic and aperiodic structure cannot be achieved by this method. By recording four-beam interference pattern into photoresist, a periodical pillar array is obtained as the sacrificial template [78], which can be used to form microlenses [79,80], optical biosensors [81], or microaxicons for beam generation [82]. The production and optical characterization of a high-quality, high-index 3D photonic crystal using MBIL and ALD was first demonstrated in 2006 [74]. A high-quality photonic crystal in amorphous TiO_2_ was produced after removal of the polymer template, showing potential applications in all-optical circuits, microcavity-based light emitters, and other microstructured photonic devices, as shown in Figure 8d. Min et al. [75] also prepared complex Pt nano-accordion structures using a combination of MBIL and ALA; these metal nanostructures have good structural stability and electrical conductivity, as shown in Figure 8e, and their cross sectional profiles can be designed by specifying the template geometry, showing applications in stretchable electronics, photonics, and nanofluidics.

PnP and MBIL methods consume less time than EBL and DLW techniques, which reduces the fabrication cost greatly. Remarkably, there is also no anisotropic volume shrinkage occurring during the calcination to remove the template in the fabrication by PnP and MBIL, providing beneficial optical properties and mechanical stability, showing potential applications for highly efficient gas sensors by utilizing high porosity and large surface area. However, the simplex and inflexible morphologies by PnP and MBIL show less flexibility and controllability, which may limit the application area.

### 3.2. Assembled Polymer Templates

**Figure 9:**
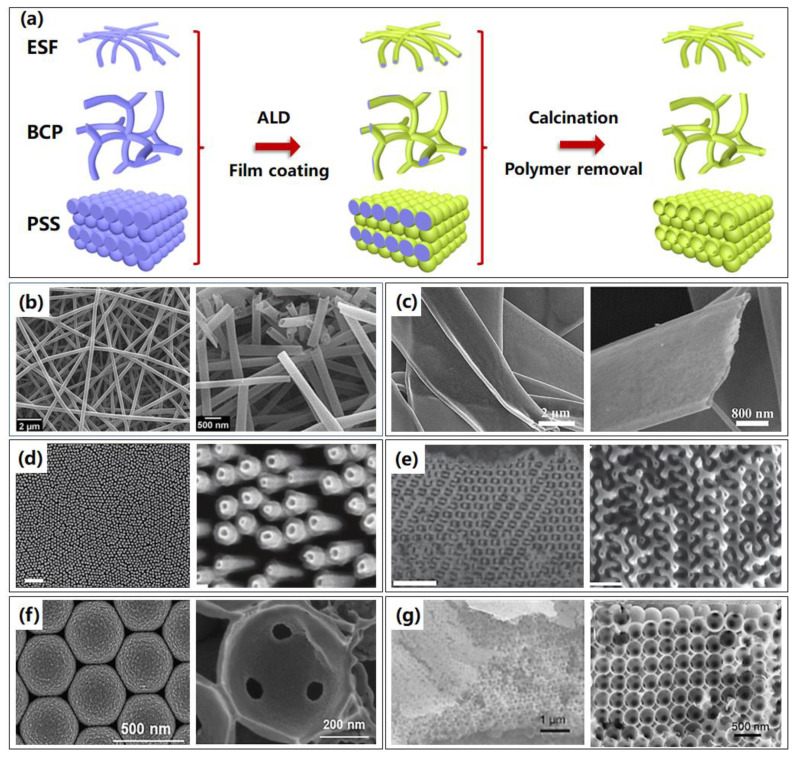
Typical assembled polymer templates are combined with ALA method to fabricate 3D nanostructures. (**a**) The illustrated process routines of 3D nanostructures based on assembled polymer templates through electrospinning fibers (ESF), blocked polymer (BCP), and polystyrene spheres (PSS) templates. (**b**) Representative SEM images of AlN hollow nanofibers based on electrospinning after calcination. Reprinted with permission from Ref. [83], Copyright 2014, with permission from authors. (**c**) Overall and cross section SEM images of belt-like BiVO_4_@ZnO heterojunction based on electrospinning after calcination. Reprinted with permission from Ref. [84], Copyright 2018 Elsevier. (**d**) Overall and enlarged SEM images of the nanorockets array based on BCP technique. Reprinted with permission from Ref. [6], Copyright 2017 WILEY-VCH Verlag GmbH & Co. KGaA, Weinheim. (**e**) SEM images of two kinds of mesoporous ZnO networks based on BCP technique. Reprinted with permission from Ref. [85], Copyright 2014 WILEY-VCH Verlag GmbH & Co. KGaA, Weinheim. (**f**) Overall and cross section SEM images of TiO_2_ inverse opals based on self-assembled PS spheres. Reprinted with permission from Ref. [86], Copyright 2020 Elsevier. (**g**) SEM images of based TiO_2_ inverse opals on self-assembled PS spheres. Reprinted with permission from Ref. [87], Copyright 2017 Elsevier.

As well as resist polymers, alternative soft templates can be the variously assembled polymer templates, which generally include a wide variety of high polymers of electrospinning fibers (ESF), blocked polymer (BCP), and self-assembly polystyrene spheres (PSS), and so forth. Obviously, ESF were fabricated by electrospinning technique, and self-assembly technique is applied to obtain the BCP and PSS templates. The 3D nanostructure fabrication processes based on ALA method through ESF, BCP, and PSS templates are similar, which firstly are the preparation of templates by corresponding techniques, followed by film deposition by ALD, and removal of templates by calcination, as shown in Figure 9a.

The major advantages of the electrospinning process are easy operating and cost-effective to implement. The wide band gap semiconductors based on electrospinning through the ALA method, such as ZnO [88,89], SnO_2_ [90,91], and TiO_2_ [88,92], including CuO [93], depicted unique surface functionalities and higher sensitivities toward ethanol, O_2_ [89,94], NO_2_ [91,92], CO [92,94], NH_3_ [88,90], H_2_ [90,95], and so forth. As a result, the 3D nanostructures based on electrospinning toward sensors were widely investigated. Haider et al. [83] reported fabrication and characterization of aluminum nitride (AlN)/boron nitride (BN) bishell hollow nanofibers through successive ALD of AlN and sequential chemical vapor deposition (CVD) of BN on electrospinning polymeric nanofibrous sacrificial templates, which are exhibited in Figure 9b. This specific structure might find potential use in composite reinforcement, chemical sensing, and gas adsorption. In addition to sensors, nanofibers based on electrospinning and ALA also displayed enhanced photocatalytic activity in water splitting [96,97]. The fabrication of BiVO_4_@ZnO heterojunction with a novel nanostructure for water splitting via electrospinning and ALA techniques were investigated [84], as shown in Figure 9c. The belt-like 3D structure exhibited enhanced photoelectrochemical performance and showed potential applications in solar cells. There are plenty of low-cost polymer materials to form polymer fiber templates using electrospinning; they could be polyacrylonitrile (PNA) [95,98], Nylon [99,100], polyvinyl acetate (PVA) [92,93], or Poly (vinylpyrrolidone) (PVP) [84,101], which show great opportunities in commercial applications.

Another important assembled polymer template for fabricating 3D nanostructures is the blocked polymer (BCP). The blocked copolymer template-assisted method, which is based on a self-assembly process, can generate well-ordered arrays with small feature sizes of 5–50 nm in dot, line, hole, or lamellar patterns, and has been extensively explored as a strategy to form periodic nanostructures [102,103]. BCP combined with ALA for wafer-scale to fabricate ultrasmall coaxial TiO_2_/Pt nanotubes was reported, which is used as a catalytic rocket with a length below 150 nm and a tubular reactor size of only 20 nm, leading to the smallest man-made rocket engine reported to date, as shown in Figure 9d [6], demonstrating abilities to efficiently power the directional transport of significantly larger passive cargo. Two other different morphologies based on the self-assembly of BCP were synthesized to fabricate mesoporous ZnO networks, as shown in Figure 9e [85]. The manufacture of ZnO-based solar cells also showed the feasibility of the integration of the 3D mesostructured ZnO networks into photovoltaic devices.

3D nanostructure could also be formed by the inverse opal (IO) structures, which possess size adjustable pore structure, large surface area, and optical properties of photonic crystals. The inverse opal nanostructures are usually fabricated using a template of silica [50] or polymer micro-spheres by the self-assembly route. Polystyrene sphere (PSS) templates are most commonly used and easily removed by calcination. Inverse opal TiO_2_ nanostructures [86] were synthesized by ALA using a template with ordered layers of PS spheres deposited on a Si substrate by spin coating, as illustrated in Figure 9f, where the IO nanostructures showed giant photocatalytic enhancement for the degradation of methylene blue than that of planar TiO_2_ films. Similarly, a TiO_2_/MoS_2_ core/shell inverse opal structure was fabricated based on ALA on a self-assembled multilayer PSS template, as presented in Figure 9g. As a 3D photonic crystal, the TiO_2_/MoS_2_ inverse opal structure exhibited obvious stopband reflecting peaks, which can be adjusted through changing the opal diameters as well as the thickness of the MoS_2_ layer [87]. The inverse opal structures could find plenty more applications in photocatalytic [104,105], solar cells [106,107], chemical sensors [108,109], surface enhanced Raman scattering (SERS) detection [110,111], etc.

The advantage of the ESF, BCP, and PSS template assisted methods should be that no complex and expensive equipment are applied to prepare the templates, as well as easy modification and feasibility of industrial-level high-volume mass production.

## 4. Comparison and Applications of 3D Nanostructures on Various Templates

The comparison of 3D nanostructures based on ALA through various rigid and soft templates are comprehensively summarized in Table 1. The templates’ fabrication and elimination methods, obtained types of 3D structures, controllability, cost, productivity, flexibility, and applications are discussed for the different templates.

### 4.1. Comparison of 3D Nanostructures on Various Templates

Rigid and soft templates with certain topographies were firstly prepared through various fabrication techniques, which could be etching, anodization, lithography, self-assembly, and even native bio-structures, which result in nanowalls, nanopores, nanotubes, nanospheres, complex 3D nanostructures, and even some customized nanostructures. Generally, the fabrications of rigid templates are relatively complicated and need excessive fabrication processes. For example, wet or dry etching and anodization are utilized to fabricate the Si and AAO templates, respectively, and commonly only nanowalls and nanopores could be achieved in the rigid templates. However, the advantages of rigid templates are that extreme high aspect ratio (hundreds:one) of nanostructures could be achieved by Si or AAO templates, which is usually unreachable for soft templates. In contrast, direct writing lithography or self-assembly are regularly applied to achieve the soft templates with various nanostructures, which are easily conducted and show enormous controllability and flexibilities in size and morphology. However, direct writing lithography is relatively time consuming and expensive, and the self-assembly process lacks controllability and flexibility.

After the sacrificial templates are prepared, essential ALD process is applied to coat or fill the templates, followed by the removal of unwanted templates leaving 3D nanostructures. For the template elimination, dry or wet etching is applied for the Si and AAO templates, which is complex and environmentally unfriendly. Another convenient and low-cost method of calcination is also applied to remove the residual templates of nanostructures based on carbon, biological, and assembled polymer templates, showing potential applications in commercial devices.

Various nanostructures could be achieved through the rigid and soft templates after the removal of the sacrificial templates. The electron and photoresist polymer templates exhibit the supreme controllability and flexibility over size and morphology, which could realize almost arbitrary customized nanostructures with high precision, including nanotubes, nanopillars, and custom structures. Especially, arbitrary custom 3D nanostructures with precise resolution and scale down to nanometers could be achieved based on ALA through electron resist, showing powerful applications in various metasurfaces. Therefore, the 3D nanostructures based on ALA method through resist polymer templates exhibit supreme controllability and flexibility. However, relatively low productivity increases the fabrication cost and inhibits the applications in commercial areas. The Si and AAO templates could also achieve various considerable nanostructures, with limited controllability and flexibility, which are generally limited to nanotubes and nanopillars. The integration with a modern conductor fabrication process demands to be developed further to fulfil modern industry need, as well as the exploitation of an environmentally friendly routine for removing the Si and AAO templates.

As to the nanostructures based on carbon and PS spheres, electrospinning fibers, BCP, and biological templates, which are usually achieved by repetition of the inflexible instinct structures, are relatively low cost and possess high productivity but lack high structure resolution, flexibility, and good controllability. As a result, it should be a comprehensive consideration to choose suitable templates and techniques for fabricating 3D nanostructures according to actual needs and conditions.

### 4.2. Applications of 3D Nanostructures on Various Templates

Along with innovating fabrication methods, expanding the application areas of 3D nanostructures is a growing demand of scientists. Due to the excellent controllability and flexibility of the ALA method based on electron resist templates, this approach provides a powerful platform to reconcile a new flexible 3D nanofabrication with versatile and multiple nanostructures towards broad potential applications in various custom applications in optical, electronic, and sensing fields, like nanophotonics, nanosensing, nanoelectronics, and nanobionics devices [40,54,112,113]. The 3D nanostructures based on AAO templates exhibit applications in plenty of different areas. For example, high aspect 3D nanostructures through AAO templates based on ALA of TiO_2_ [114,115,116,117], ZnO [118,119,120], and HfO_2_ [121,122] were achieved and further investigated, revealing potential applications in various advanced devices, including fluorescence, sensors, detectors, 3D batteries and supercapacitors, and so on.

Furthermore, the utilizations of 3D nanostructures fabricated by ALA method through other rigid and soft templates in catalytic, sensing, and engineering devices are attracting more attention, as illustrated in Table 1. For example, many 3D nanostructures based on ALA method through different sacrificial templates, with the materials of TiO_2_, ZnO, SnO, et al., are photoactive, and thus their practical applications can be mostly found in the fields of photovoltaics [123,124], photoelectrolysis [115,125], photoelectrode [126], and photonic crystals [87,127]. Furthermore, recalling the well-known catalytic activity, these 3D nanostructures fabricated based on various templates, like biological, ES fibers, BCP, and PS sphere templates, provide us with an attractive means for fabricating efficient devices with high-surface-area surfaces for catalysis [128,129,130]. In addition, another field where high surface area is beneficial is sensor application. Various sensors with 3D nanostructures, like gas [131,132] and biological [133,134,135] sensors, have been investigated and exhibit excellent stability and response. Up to now, the applications of 3D nanostructures based on various rigid and soft templates have covered many other fields and attracted more and more researchers’ attentions, like mechanical [57,136,137], electrical [40,138], optical [29,68], and flexible devices [139,140,141]. Certainly, all these applications are mass-produced only if reliable, controllable, and low-cost nanofabrication processes of 3D nanostructures are achieved and further improved, but they provide a promising routine for future commercial industry.

## 5. Conclusions and Outlook

Considering the specialty and importance of 3D nanostructures in excellent functionalities over planar nanostructures, 3D nanostructures have undergone rapid development in recent years, but no mature strategies for mass production exist thus far. ALD assisted 3D assembly through various sacrificial templates offers a promising approach to smoothly implementing the fabrication of 3D nanostructures. In this review, we have systemically addressed the fabrication strategies for 3D nanostructures based on the ALA method through various rigid and soft templates.

Many hard and soft templates with abundant geometries can be used by ALA method to fabricate most 3D nanostructures; key factors including reasonable critical dimension of ALD, the compatibility with the ALD process, and acceptable time consumption should be fully considered as golden rules to judge the feasibility of using the ALA method.

The goal for the development of 3D nanostructures by ALA method based on sacrificial templates is to fabricate commercial devices that will be available in the market, but this nanofabrication strategy still needs great improvement and advancement. 3D nanostructure-based devices still face many challenges, such as achieving a large scale, lost-cost, uniform, high-productivity and precise controllability. These challenges cannot be fully addressed with ALA methods based on various sacrificial templates thus far. Furthermore, integration with traditional semiconductor techniques is still the orientation of 3D nanostructure development. With the recent development of nanofabrication techniques, new fabrication strategies based on ALA method are expected to emerge; we are confident that such nanofabrication platforms with reduced cost, high productivity, precise controllability, and environmentally friendly for 3D nanostructure devices will eventually be commercially available, although great efforts are still needed.

## Figures and Tables

**Table 1:** Comparison of nanostructures based on rigid and soft templates. ^(a)^ L = low, M = medium, H = high; ^(b)^ ● Optical devices, ★ Electronic devices, ■ Engineering devices, ◆ Sensing devices, ▲ Catalytic device.

Template		Template Fabrication Technique	Template Elimination Technique	Obtained 3D Nanostructures	Controllability ^(a)^	Cost	Productivity	Flexibility	Application ^(b)^
Rigid templates	Si	Etching (Dry, wet)	Etching (Dry, wet)	Nanotubes, Nanopillars	M	M	L	M	●★◆
	AAO	Anodization	Wet etching	Nanotubes, Nanopillars	M	M	M	M	●★◆
	Biological structure	Native	Calcination	Replica of biological structures	L	L	H	L	●◆▲
	Carbon	CVD	Calcination	Nanotubes Nanospheres	L	L	M	L	●▲
	Electron resist	EBL	O_2_ plasma/Dissolution	Nanotubes, Nanopillars, Custom structures	H	H	L	H	●★■◆▲
Soft templates	Photoresist	DLW/PnP/MBIL	O_2_ plasma	Nanotubes, Nanopillars, Custom structures	H	M	M	H	●★■◆▲
	Fiber	ES	Calcination	Nanofibers	L	L	H	L	★◆▲
	BCP	SA	Calcination	3D networks	M	L	H	M	●★■◆▲
	PS sphere	SA	Calcination	Inverse-opals	L	L	H	L	●◆▲

## Data Availability

Not applicable.
